# Perceptions and Knowledge Regarding Medical Situations at the End of Life among Older Adults in Switzerland

**DOI:** 10.1089/jpm.2022.0057

**Published:** 2022-12-30

**Authors:** Clément Meier, Sarah Vilpert, Gian Domenico Borasio, Jürgen Maurer, Ralf J. Jox

**Affiliations:** ^1^ Faculty of Biology and Medicine (FBM), Swiss Centre of Expertise in the Social Sciences (FORS), University of Lausanne, Lausanne, Switzerland.; ^2^ Faculty of Business and Economics (HEC), Swiss Centre of Expertise in the Social Sciences (FORS), University of Lausanne, Lausanne, Switzerland.; ^3^ Palliative and Supportive Care Service, Lausanne University Hospital and University of Lausanne, Lausanne, Switzerland.; ^4^ Faculty of Business and Economics (HEC), University of Lausanne, Lausanne, Switzerland.; ^5^ Institute of Humanities in Medicine, Lausanne University Hospital and University of Lausanne, Lausanne, Switzerland.

**Keywords:** end of life, knowledge, older adults, perceptions, population-based study, Switzerland

## Abstract

**Background:**

Perceptions and knowledge regarding end-of-life health and health care can influence individuals' advance care planning, such as the completion and content of advance directives.

**Objectives:**

To assess older adults' perceptions of medical end-of-life situations in Switzerland along with their accuracy and corresponding associations with sociodemographic characteristics.

**Design:**

This is an observational study.

**Setting/study subjects:**

: A nationally representative sample of adults aged 58 years and older who participated in wave 8 (2019/2020) of the Swiss part of the Survey of Health, Ageing, and Retirement in Europe (cooperation rate: 94.3%).

**Measurements:**

Subjective likelihood of 11 end-of-life situations on a 4-point scale: very unlikely (0–25%), rather unlikely (26%–50%), rather likely (51%–75%), and very likely (76%–100%).

**Results:**

Older adults' perceptions of end-of-life medical situations in Switzerland were rather heterogeneous and often inaccurate. Study subjects overestimated the success of cardiopulmonary resuscitation, the utility of a fourth-line chemotherapy, of hospital admission for pneumonia for patients with advanced dementia, and for artificial nutrition and hydration in the dying phase, while underestimating the effectiveness of pain management in this situation. Less than 28% of older adults correctly assessed the likelihood of dying in a nursing home, hospital, or at home, respectively. Inaccurate views were more frequent in men (*p* < 0.01) and individuals with financial difficulties (*p* < 0.05), whereas adults aged 75+ years (*p* < 0.01) and respondents from the German-speaking part of Switzerland (*p* < 0.01) had more accurate perceptions.

**Conclusions:**

The wide variation and low accuracy of end-of-life perceptions suggest considerable scope for communication interventions about the reality of end-of-life health and health care in Switzerland.

## Introduction

Population aging and an increasing medicalization of older age, especially at the end of life, have led to major changes in the social and health care contexts in which death occurs.^1^ These trends have led to a higher predictability of the end of life and a greater importance of considering individual preferences for end-of-life care.^2^ These circumstances have stimulated the development and implementation of advance care planning as a tool to better align end-of-life care with individuals' wishes for their end of life, which often concern diverse aspects of quality of life beyond health care.^3,4^ Thus, individuals can decide about their end-of-life medical treatments, whereas direct contact with death has generally been evicted from our societies. As a result, individuals are asked to make choices about situations they have little knowledge about.

The decision to engage in advance care planning and the content of advance care plans are likely to depend on people's perceptions and knowledge regarding the reality of end-of-life health care in the setting in which they live.^5^ Key aspects to consider when planning for the end of life include the risk to suffer from dementia in old age, legal liability for medical decision making at the end of life, the potential utility of different types of medical interventions at the end of life, and considerations of place of death. In Switzerland, dementia is the third leading cause of death^6^; patients with dementia tend to be a burden for their families that have to make medical decisions on their behalf^7^; detecting such illness at an early stage may allow individuals to state their preferences for medical treatments.^8,9^

Perceptions and knowledge play a crucial part in decision making regarding medical treatment; common sources of distress reported by patients are pain management,^10^ even though it is well treatable by specialists,^11^ artificial nutrition, and hydration treatments viewed as either compassionate or invasive,^12^ cardiopulmonary resuscitation in a cardiac arrest that is usually considered as a successful medical intervention,^13^ and the use of medically inappropriate treatments.^14^ Finally, individuals receive different types of care, and their end-of-life experience varies depending on whether they die in a nursing home, a hospital, or at home.^15,16^

The complexity of end-of-life situations and the potentially negative consequences of an uninformed or misinformed decision on the quality of dying and death emphasize the need for early communication on end-of-life medical issues to avoid misunderstanding or misrepresentation. Communication is a central part of end-of-life care; patients and family caregivers need information about the illness and its progression.^17^ Having an enlightened view of the reality of end-of-life care is, therefore, a key input into advance care planning, and reducing misconceptions and knowledge gaps about likely end-of-life outcomes may enable older adults to prepare and manage their end of life more effectively.^18^

Despite the general importance of perceptions of end-of-life realities for potential advance care planning, little is known about the perceptions of common end-of-life situations and their accuracy among older adults in the general population. To fill this knowledge gap, we used nationally representative data on adults aged 58 years and older in Switzerland to assess their perceptions of important aspects of end-of-life health and health care along with their accuracy in the Swiss context. We specifically explored individuals' perceptions and potential misrepresentations about frequent health and medical end-of-life situations concerning cognitive impairment, life-sustaining measures, medically inappropriate treatments, and place of death.

## Methods

### Study design and sample

We used data from the Survey of Health, Ageing and Retirement in Europe (SHARE), a European biennial population-based cohort study of adults aged 50 years and older and their partners, which collects information on health, socioeconomic status, and social networks.^19,20^ The data set combines internationally harmonized face-to-face interviews and national self-administered paper-and-pencil questionnaires handed out to participants at the end of the interview. Our analytical data set includes questions from the Swiss paper-and-pencil questionnaire on end-of-life issues and sociodemographic variables merged from the face-to-face interview, both from wave 8 of the SHARE study fielded between October 2019 and the beginning of March 2020. The IRB approval: CER-VD: 66/14 (2014).

**APPENDIX FIG. A1.**
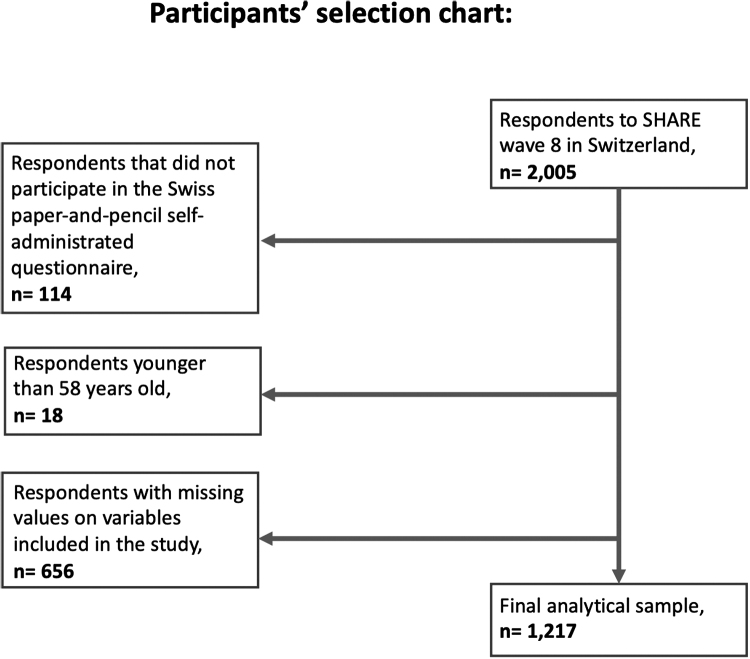
Participants' selection chart.

The last refreshment sample of SHARE Switzerland took place in 2011; thus, the study only includes target respondents aged 58 years and older to be nationally representative. The main interview from wave 8 of SHARE included 2005 Swiss respondents; among them, 94.3% also completed the paper-and-pencil questionnaire (*n* = 1891). After restricting the sample to participants from the age of 58 years onward and deleting all observations with missing information on at least one item used in the analysis, the total number of individuals included was 1217 (Appendix Figure A1).

### Measures

#### Outcome variables

##### Perceived frequency of different end-of-life situations

The questionnaire included 11 frequent end-of-life health and health care situations regarding cognitive impairment, medical treatment, and place of death (Appendix Table A1). Respondents had to evaluate the frequency of occurrence of the 11 situations on a 4-point scale: 1 = very unlikely (0–25%), 2 = rather unlikely (26%–50%), 3 = rather likely (51%–75%), and 4 = very likely (76%–100%). The accuracy of perceptions of different end-of-life situations is defined by dichotomized variables where 1 indicates a correct answer and 0 an incorrect answer.

### Independent variables

#### Sociodemographic covariates

To assess sociodemographic differences in perceived likelihood of different end-of-life situations and their accuracy, our statistical models include information on gender (0 = male, 1 = female), age group (58–64 , 65–74, and 75+ years), and education level, which was grouped into three categories based on the International Standard Classification of Education (ISCED) of 2017^21^ (low = ISCED levels 0–1–2, secondary = ISCED levels 3–4, tertiary = ISCED levels 5–6). We also used information on the three main linguistic regions of Switzerland based on the language completion of the questionnaire (German, French, or Italian).

Our measure of partnership status considered all types of partnership rather than just focusing on formal marriage (0 = has a partner, 1 = has no partner). Respondents' perceived financial situation was measured based on the question: “Is your household able to make ends meet?” with permissible answers being recoded into three groups (1 = easily, 2 = fairly easily, and 3 = with difficulty). We also used information on whether individuals lived in an urban or rural area (0 = urban, 1 = rural) and respondents' self-rated health coded on a 3-point scale (1 = poor/fair health, 2 = good health, and 3 = very good/excellent health).

### Statistical analysis

We calculated respondents' weighted proportion estimation per category of probabilities of the 11 end-of-life health and health care situations and indicated the correct answer based on recent studies from the literature (Appendix Table A2). The sample was calibrated using cross-sectional weights provided in the SHARE data.^22^ We then determined the partial associations between the 11 end-of-life health and health care situations and the individuals' characteristics using unweighted interval regressions. Finally, we presented 11 unweighted probit regressions of variables assessing the degree of accuracy of respondents' perceptions on each situation (0 = incorrect answer, 1 = correct answer) on individuals' sociodemographic characteristics.

In addition, we also calculated a score regarding the accuracy of respondents' perceptions; the score adds 1 point for a right answer and 0; otherwise, the maximum possible value of the score is 11, and the minimum is 0. We did an ordinary least squares (OLS) regression of the score on respondents' characteristics. Moreover, since this was an exploratory study, we did not adjust for multiple comparisons. All estimations were performed using STATA/SE 17.0 software (STATA Corporation, College Station, TX, USA), with standard errors clustered at the household level.

## Results

Table 1 presents the key characteristics of our sample. The proportion of women was 48%, the median age was 65 years old, with 22% of the respondents older than 75 years. Most respondents had a partner (74%). Concerning the linguistic regions, 72% were from the German-speaking part, 25% from the French-speaking region, and 3% from the Italian-speaking part. The majority had a secondary level of education (66%) and 12% a lower level. Most of the respondents reported that it was “easy” (58%) or “fairly easy” (31%) to make ends meet at the end of the month. Moreover, a majority of 59% lived in a rural area. Finally, most of the respondents self-reported to have “good” or “excellent health” (86%).

**Table 1.** Characteristics of the Study Population, Adults Aged 58+ Years, Survey of Health, Ageing and Retirement in Europe Switzerland, 2019/2020 (*N* = 1217)

	Unweighted	Weighted	
	Obs	%	CI
Gender			
Male	592	52	47.7–57.2
Female	625	48	42.8–52.5
Age groups			
58–64 Years	225	49	43.5–55.2
65–74 Years	564	29	25.6–33.2
75+ Years	428	22	18.6–24.6
Partnership status			
Has a partner	955	74	69.2–79.1
No partner	262	26	20.9–30.8
Linguistic regions			
German	881	72	66.6–77.1
French	301	25	20.5–30.9
Italian	35	3	1.5–4.1
Education			
Basic	172	12	9.1–15.1
Secondary	785	66	60.3–70.6
Tertiary	260	22	18.1–27.8
Make ends meet			
Easily	685	58	52.1–62.8
Fairly easily	387	31	26.2–36.1
With difficulty	145	11	8.6–15.4
Living area			
Urban	549	41	35.3–46.1
Rural	668	59	53.9–64.7
Self-rated health			
Poor/fair health	205	14	11.3–18.3
Good health	499	38	32.5–42.9
Excellent health	513	48	42.3–53.7

Unweighted and weighted number of observations for the whole sample.

**FIG. 1.**
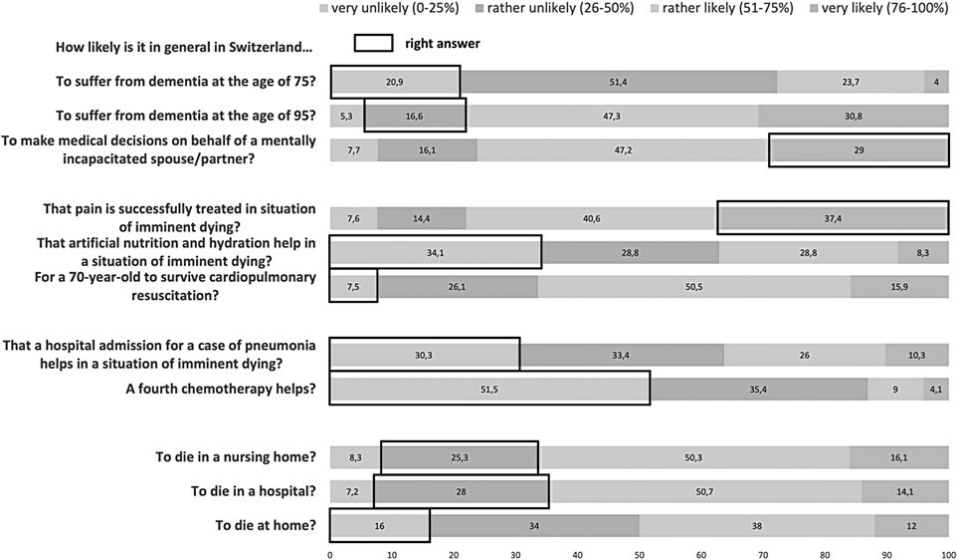
Percentage of respondents per categories of each end-of-life representations, adults aged 58+ years, SHARE Switzerland, 2019/2020, *n* = 1217. SHARE, Survey of Health, Ageing and Retirement in Europe.

Figure 1 shows respondents' perceived likelihood of the 11 end-of-life health and health care situations with the correct answer marked by a rectangle. Regarding the likelihood of suffering from dementia at higher ages, respondents correctly perceived this likelihood to be larger at age 95 years compared with age 75 years but generally overestimated the prevalence of dementia at both ages. Although the majority of respondents reported it to be “rather likely” or “very likely” to be asked to make medical decisions on behalf of their spouse or partner in case of their decisional incapacity and the absence of an advance directive, only less than one-third of respondents selected the correct category.

Individuals overestimated the success of cardiopulmonary resuscitation, the utility of a fourth-line oncological chemotherapy, the benefit of hospital admission for pneumonia in advanced dementia, and the effect of artificial nutrition and hydration in the dying phase but underestimated the effectiveness of pain management in this context. Concerning the likelihood of different places of death, older adults in general correctly think that deaths in Switzerland mostly occur in nursing homes and hospitals, with fewer people dying at home. However, most respondents generally overestimated the likelihood of the different places of death, with <28% choosing the correct probability for dying in a nursing home, hospital, or at home, respectively.

Table 2 gives the partial associations between each respondents' perceptions of end-of-life health and health care situations and the individuals' characteristics. Overall, the results showed significant associations of perceptions with respondents' gender, age, education level, linguistic region, and self-rated health.

**Table 2.** Interval Regressions of the End-of-Life Representations' Items on the Covariates, Adults Aged 58+ Years, Survey of Health, Ageing and Retirement in Europe Switzerland, 2019/2020 (*N* = 1217)

	Dementia 75	Dementia 95	Decisions	Pain	Artificial	Cardiopulmonary	Pneumonia	Chemotherapy	Nursing	Hospital	Home
Gender (male)											
Female	2.71^*^ (1.15)	3.62^**^ (1.22)	2.77^*^ (1.27)	1.99 (1.31)	−5.77^***^ (1.40)	0.21 (1.22)	−3.73^**^ (1.43)	0.73 (1.19)	4.32^***^ (1.17)	4.96^***^ (1.14)	−4.02^***^ (1.22)
Age group (58–64 years)											
65–74 Years	−1.60 (1.48)	−1.07 (1.72)	−1.64 (1.71)	−0.67 (1.83)	0.36 (2.00)	−2.77 (1.69)	−3.36 (1.94)	−0.05 (1.74)	−3.12 (1.63)	0.35 (1.63)	−2.43 (1.75)
75+ Years	−7.90^***^ (1.72)	−6.14^**^ (1.88)	−6.64^***^ (1.89)	−4.93^*^ (2.02)	−3.53 (2.09)	−3.81^*^ (1.88)	−5.79^**^ (2.12)	−1.80 (1.87)	−5.98^***^ (1.77)	−0.90 (1.79)	0.12 (1.92)
Partnership status (has a partner)											
No partner	0.11 (1.51)	−3.70^*^ (1.62)	−3.16 (1.64)	0.28 (1.68)	−0.08 (1.79)	−2.02 (1.67)	−0.73 (1.86)	−2.11 (1.56)	1.08 (1.57)	−1.62 (1.53)	−0.15 (1.64)
Linguistic regions (German)											
French	−0.73 (1.31)	1.65 (1.49)	1.78 (1.44)	−0.29 (1.52)	7.26^***^ (1.66)	3.22^*^ (1.47)	3.43^*^ (1.68)	−2.93^*^ (1.44)	6.90^***^ (1.46)	2.52 (1.37)	−5.54^***^ (1.50)
Italian	3.03 (4.36)	9.10^**^ (3.12)	−3.72 (4.93)	4.75 (3.60)	13.41^**^ (4.77)	4.87 (4.33)	0.03 (4.85)	6.97 (4.87)	12.61^***^ (2.60)	6.18 (3.56)	−5.72 (3.38)
Education (low)											
Secondary	−1.17 (1.87)	1.49 (2.07)	−1.07 (2.06)	0.85 (2.10)	−1.19 (2.14)	1.32 (1.98)	−1.37 (2.24)	−4.76^*^ (2.17)	0.57 (1.90)	2.88 (1.92)	−1.14 (1.87)
Tertiary	−3.91 (2.07)	4.90^*^ (2.33)	2.70 (2.32)	6.47^**^ (2.34)	−2.00 (2.54)	2.16 (2.31)	−4.25 (2.67)	−5.68^*^ (2.45)	1.39 (2.15)	1.34 (2.22)	−1.83 (2.19)
Make ends meet (easily)											
Fairly easily	−0.75 (1.32)	2.12	0.75 (1.45)	−1.72 (1.56)	1.21 (1.57)	0.18 (1.47)	−0.06 (1.64)	−0.22 (1.38)	−0.72 (1.34)	−0.45 (1.28)	1.96 (1.44)
		(1.45)									
With difficulty	2.74 (2.29)	0.43	−0.82 (2.13)	−2.02 (2.25)	2.60 (2.52)	0.61 (2.10)	3.95 (2.42)	1.24 (2.16)	−1.93 (2.21)	−3.21 (2.00)	2.56 (2.16)
		(2.41)									
Living area (urban)											
Rural	−0.22 (1.18)	1.11	0.48 (1.33)	−0.31 (1.36)	0.17 (1.44)	1.05 (1.28)	0.28 (1.49)	−1.02 (1.27)	−0.05 (1.20)	−1.07 (1.17)	−0.34 (1.29)
		(1.28)									
Self-rated health (bad health)											
Good health	1.53 (1.71)	−0.59	0.93 (1.87)	4.13^*^ (2.02)	0.25 (2.12)	0.04 (1.97)	−2.30 (2.16)	−2.15 (1.92)	−0.48 (1.81)	−1.37 (1.80)	3.68^*^ (1.86)
		(1.97)									
Excellent health	−0.71 (1.78)	−0.08	3.49 (1.97)	5.55^**^ (2.09)	0.90 (2.17)	2.35 (2.05)	−2.45 (2.24)	−4.14^*^ (1.97)	−1.95 (1.92)	−1.25 (1.82)	5.97^**^ (1.94)
		(1.98)									
Observations	1217	1217	1217	1217	1217	1217	1217	1217	1217	1217	1217

This table reports the estimates for the interval regressions of the end-of-life representations' items on the covariates. Standard errors in parentheses have the following significance levels ^*^p < 0.05, ^**^p < 0.01, ^***^p < 0.001. Concerning the interpretation, for instance, the number on top-left in bold means that compared with men, women have 2.71% more chances to say that it is likely in general to suffer from dementia at the age of 75 years old in Switzerland.

Table 3 presents the partial associations between the degree of accuracy of respondents' perceptions on the 11 end-of-life health and health care situations and individuals' sociodemographic characteristics. The results from the score regarding the accuracy showed that, overall, compared with men, women were more likely to pick the correct answer category (*p* < 0.01). Then, compared with the younger group, respondents older than 75 years were more likely to give accurate answers (*p* < 0.01). In addition, compared with the French-speaking part of Switzerland, respondents from the German-speaking region were more likely to answer correctly (*p* < 0.01). Finally, respondents reporting that it is difficult to make ends meet compared with those with financial facilities were less likely to choose the right answer (*p* < 0.05).

**Table 3.** Probit Regressions of the Correct Answer on the End-of-Life Representations' Items on Covariates, Adults Aged 58+ years, Survey of Health, Ageing and Retirement in Europe Switzerland, 2019/2020 (*N* = 1217)

	Score	Dementia 75	Dementia 95	Decisions	Pain	Artificial	Cardiopulmonary	Pneumonia	Chemotherapy	Nursing	Hospital	Home
Gender (male)												
Female	0.24^**^ (0.09)	−0.04 (0.02)	−0.06^**^ (0.02)	0.05^*^ (0.03)	0.08^**^ (0.03)	0.13^***^ (0.03)	0.02 (0.02)	0.11^***^ (0.03)	−0.02 (0.03)	−0.05^*^ (0.03)	−0.05^**^ (0.02)	0.06^*^ (0.03)
Age group (58–64 years)												
65–74 Years	0.05 (0.13)	−0.00 (0.03)	−0.03 (0.03)	−0.00 (0.04)	−0.02 (0.04)	−0.01 (0.04)	0.01 (0.02)	0.08^*^ (0.03)	−0.02 (0.04)	−0.03 (0.03)	−0.03 (0.02)	0.10^**^ (0.04)
75+ Years	0.41^**^ (0.14)	0.23^***^ (0.04)	0.02 (0.03)	−0.10^*^ (0.04)	−0.07 (0.04)	0.04 (0.04)	0.05 (0.03)	0.13^**^ (0.04)	0.03 (0.04)	0.04 (0.04)	−0.01 (0.03)	0.05 (0.04)
Partnership status (has a partner)												
No partner	0.09 (0.12)	0.01 (0.03)	0.01 (0.03)	−0.02 (0.03)	0.01 (0.04)	0.03 (0.03)	0.05^*^ (0.02)	0.02 (0.03)	0.05 (0.04)	−0.06 (0.03)	0.05^*^ (0.02)	−0.07^*^ (0.03)
Linguistic regions (German)												
French	−0.35^**^ (0.11)	0.03 (0.03)	−0.05^*^ (0.02)	−0.00 (0.03)	−0.05 (0.03)	−0.12^***^ (0.03)	−0.02 (0.02)	−0.06^*^ (0.03)	0.06 (0.04)	−0.14^***^ (0.03)	0.00 (0.02)	−0.01 (0.03)
Italian	−0.22 (0.39)	0.08 (0.08)	−0.10^*^ (0.04)	−0.03 (0.08)	0.06 (0.08)	−0.20^**^ (0.08)	0.03 (0.05)	0.03 (0.09)	−0.09 (0.08)	−0.15^*^ (0.06)	−0.01 (0.04)	0.17 (0.09)
Education (low)												
Secondary	−0.11 (0.14)	0.00 (0.04)	−0.06 (0.03)	−0.07 (0.04)	0.00 (0.04)	0.01 (0.04)	−0.00 (0.03)	−0.01 (0.04)	0.03 (0.04)	−0.01 (0.04)	−0.06^*^ (0.03)	0.04 (0.04)
Tertiary	0.15 (0.17)	−0.00 (0.04)	−0.07 (0.04)	−0.01 (0.05)	0.09 (0.05)	0.01 (0.05)	−0.03 (0.03)	0.06 (0.05)	0.02 (0.05)	0.03 (0.05)	−0.05 (0.03)	0.10^*^ (0.05)
Make ends meet (easily)												
Fairly easily	−0.11 (0.11)	0.00 (0.03)	−0.01 (0.02)	−0.00 (0.03)	−0.04 (0.03)	−0.04 (0.03)	−0.00 (0.02)	−0.01 (0.03)	−0.00 (0.03)	0.00 (0.03)	−0.01 (0.02)	−0.01 (0.03)
With difficulty	−0.34^*^ (0.16)	−0.01 (0.04)	−0.00 (0.04)	−0.03 (0.04)	−0.07 (0.05)	−0.05 (0.05)	−0.03 (0.02)	−0.09^*^ (0.04)	−0.02 (0.05)	−0.04 (0.04)	0.01 (0.03)	−0.02 (0.05)
Living area (urban)												
Rural	0.04 (0.10)	−0.01 (0.02)	−0.01 (0.02)	−0.00 (0.03)	−0.01 (0.03)	−0.00 (0.03)	−0.02 (0.02)	−0.00 (0.03)	0.01 (0.03)	0.03 (0.03)	0.02 (0.02)	0.03 (0.03)
Self-rated health (bad health)												
Good health	−0.00 (0.14)	−0.03 (0.03)	0.01 (0.03)	0.01 (0.04)	0.02 (0.04)	−0.02 (0.04)	−0.05 (0.03)	0.01 (0.04)	0.00 (0.04)	−0.01 (0.04)	−0.01 (0.02)	0.08^*^ (0.04)
Very good/excellent health	0.16 (0.14)	0.00 (0.04)	−0.00 (0.03)	0.09^*^ (0.04)	0.07 (0.04)	−0.01 (0.04)	−0.05 (0.03)	0.04 (0.04)	0.06 (0.04)	−0.04 (0.04)	−0.01 (0.02)	0.03 (0.04)
Constant	2.95^***^ (0.24)											
Observations	1217	1217	1217	1217	1217	1217	1217	1217	1217	1217	1217	1217

This table reports on the first column an OLS regression of the score regarding the accuracy of each individual's perception; the score adds 1 point for a right answer and 0; otherwise, the maximum possible value of the score is 11, and the minimum is 0. The rest of the table are probit regressions of the binary variable correct answers on the end-of-life representations' items on the covariates. Average marginal effects and standard errors in parentheses have the following significance levels ^*^p < 0.05, ^**^p < 0.01, ^***^p < 0.001. The interpretation of the number in bold means that compared with men, women are 6% less likely to give the correct answer on the likelihood to suffer from dementia at the age of 95 years old in Switzerland.

## Discussion

To the best of our knowledge, our study is the first nationally representative population-based study to assess older adults' perceptions and accuracy of health and health care at end-of-life situations in Switzerland. We found considerable heterogeneity in older adults' end-of-life representations with overestimation of the prevalence of dementia, rather low level of accuracy regarding pain management effectiveness and artificial nutrition and hydration utility in a situation of imminent death, unrealistic expectations of the survival rate after cardiopulmonary resuscitation at older ages, and high percentages of inaccuracy regarding the place of death in Switzerland. In addition, our findings show differences in perceptions of end-of-life health and health care situations and their accuracy among some population groups in Switzerland.

### Cognitive impairment

In Switzerland, the prevalence estimate of dementia is <5% for individuals aged <75 years old and ∼45% for adults of 95 years old.^23^ Most respondents overestimated the likelihood to suffer from dementia at the age of 75 and 95 years. This result may reflect a relative ignorance of dementia in the general population.

The frequent misrepresentations of dementia result in stigmatization and add barriers to early detection and treatment,^24^ which may disadvantage individuals affected by dementia regarding their autonomy at the end of life, and shift the weight of treatment decisions to their partner or family. Indeed, in Switzerland, the federal law stipulates that if a person does not have decision-making capacity and has not designated a proxy in the advance directive, the person who has to decide is the partner of the patient.^25^

### Medical treatment

Irrespective of the effectiveness of medical treatment, a common fear is that dying patients would suffer from severe pain, negatively affecting the quality of their end of life.^26^ In our study, respondents of the older aged group were the most skeptical regarding successful treatment of pain in a situation of imminent dying, whereas individuals with a higher level of education and better self-rated health were the most optimistic regarding chances of effective pain management.

It has been reported that patients and family members often request health care specialists to administer oral nutrition and fluids continuously.^27,28^ Yet, artificial nutrition and hydration during the dying phase seem not to benefit patients.^29^ In our sample, however, many respondents believed that artificial nutrition and hydration are unlikely to help in a situation of imminent dying. This belief was stronger for women, consistent with other studies showing that women prefer to refrain from treatments that could deteriorate their quality of life.^30–32^

Differences within linguistic regions also appeared; respondents from German-speaking Switzerland, compared with the French-speaking and Italian-speaking parts, stated less often that artificial nutrition and hydration help in imminent dying. The variation within linguistic regions is potentially due to differences in cultural or religious beliefs.^33^

Finally, in our sample, most respondents believed it is likely for a 70 years old to survive cardiopulmonary resuscitation outside of a hospital. This result shows that older adults in Switzerland had exceedingly unrealistic expectations because the survival rate in these conditions is <8%.^34^ The overoptimistic perspective adds difficulties for health care providers who have to communicate with patients and families and may lead to inappropriate medical decision making.^35^

### Medically inappropriate treatments

Infectious diseases often precipitate the death of patients who are already weakened by multiple morbidities.^36^ A situation emblematic of this sequence is that of patients with advanced dementia living in a nursing home who develop an acute infection such as pneumonia.^37^ In that case, maintaining these patients in the nursing home and administering oral antibiotics there instead of transferring them to a hospital are more beneficial regarding quality of dying and provide considerable cost savings.^38^ In our study, most respondents understood the unsuitability of a patient's hospitalization in such a situation.

Most respondents gave an accurate answer concerning the probability that a fourth-line chemotherapy would help a patient with advanced cancer after three different lines of chemotherapy failed. The chances of surviving such a medical treatment are ∼13.6% in Switzerland.^39–41^ However, a portion of older adults living in Switzerland believed that medical action in these two end-of-life situations that can be considered aggressive treatments would benefit the patient. These beliefs show the need to communicate well about common end-of-life situations to ensure that everybody can make an informed assessment.

### The place of death

Although most Swiss residents prefer to die at home,^42^ 44% do in fact die in a nursing home, 37% in a hospital, and only 19% at home or elsewhere,^43^ with an increasing trend toward dying in institutions.^44^ In our study, most respondents systematically overestimated the likelihood of dying in a nursing home, a hospital, or at home. In addition, for many respondents, the addition of the three percentages was higher than 100, which showed a misunderstanding regarding the question or a lack of attention.

The percentage of misrepresentation was higher for the likelihood of dying at home; this is certainly influenced by the fact that home remains the ideal place of death for a large proportion of the population.^45^ Then, respondents with poor health compared with those self-reporting good or excellent health indicated a lower probability of dying at home, maybe because older individuals and those with poor health are more likely to understand the limitations of home care services.^46^ Knowing the likelihood of dying at home would allow individuals to adapt and prepare multiple aspects of their life, such as the finances, family organization, and care, to increase the chances of staying home at the end of their lives.

### Limitations

Our study has some limitations. First, using a combination of qualitative and quantitative labels for the answer categories may have led to confusion among respondents. Some respondents may have just used either the qualitative or the quantitative labels when constructing their answers, which could result in measurement error and limitations in the comparability of answers with corresponding estimates from the literature. Second, respondents may have insufficiently understood the concepts of probability used in the construction of answer categories, despite having been asked similar subjective probability questions several times throughout the SHARE study.

Third, although SHARE makes every effort to remain representative of the older population in Switzerland, selective attrition from the study, notably among the oldest and most frail respondents, may result in some sample selection. Fourth, item nonresponse and corresponding sample selection effects may have biased our analysis, although our findings seem largely robust to deviations from our complete case analysis, such as the use of item-specific subsamples for each outcome.

## Conclusion

The overall qualitative patterns of perceptions of many aspects of end-of-life situations of older adults in Switzerland seem largely consistent with current reality, even if their exact quantitative assessments are often rather inaccurate. Specifically, although perceptions of the success of some medical treatments seemed largely in line with available data, perceptions of the prevalence of dementia among the oldest or of the chances of success of out-of-hospital cardiopulmonary resuscitation in an older person are far from reality.

Furthermore, perceptions often vary considerably across individuals, which highlights significant knowledge gaps regarding end-of-life health and health care realities in Switzerland in parts of the population. Indeed, women, individuals of older age groups, and the better off appeared to have more accurate perceptions of end-of-life situations. Nonetheless, our study reveals significant misrepresentations of end-of-life realities among older adults in Switzerland that may lead to unrealistic expectations regarding end-of-life care, result in the disappointment of patients and families, and compromise the quality and reliability of advance care planning.

## Data Sharing

This article uses data from Börsch-Supan, A. (2020). Survey of Health, Ageing and Retirement in Europe (SHARE) Wave 8. Release version: 1.0.0. SHARE-ERIC. Data set. DOI: 10.6103/SHARE.w8.100. Study data already deidentified are available to the scientific community upon submitting a data requestion application to the SHARE study.

## Appendix

**Appendix Table A1.** End-of-Life Medical Situation Questions

People have representations of end-of-life medical situations. We would like to know yours. We would like to know whether you think that the situations described hereunder and related to end of life are very unlikely (0–25%), rather unlikely (26%–50%), rather likely (51%–75%), or very likely (76%–100%). **If you do not know, please give us your best estimate.** *Example: “In your opinion, what are the chances that it is snowing tomorrow?” If you tick “very likely,” you consider that the chances that it is snowing tomorrow range between 76% and 100%.* In your opinion, how likely is it in general in Switzerland… Answer categories: “very unlikely (0–25%),” “rather unlikely (26%–50%),” “rather likely (51%–75%),” “very likely (76%–100%).” 1. To suffer from dementia at the age of 75 years? **(dementia 75)** 2. To suffer from dementia at the age of 95 years? **(dementia 95)** 3. In the absence of advance directives, to be asked to make medical decisions concerning your spouse/partner should he/she become severely ill and unable to make decisions? **(decisions)** 4. That pain (of any origin) can be successfully treated in a situation of imminent dying? **(pain)** 5. That artificial nutrition and hydration helps in a situation of imminent dying? **(artificial)** 6. For a 70 years old to survive until hospital discharge from a cardiopulmonary resuscitation performed outside of a hospital after a cardiac arrest? **(cardiopulmonary)** 7. That a hospital admission in the case of pneumonia helps for a patient in a situation of imminent dying due to advanced dementia living in a nursing home? **(pneumonia)** 8. That a fourth-line chemotherapy helps a patient with advanced cancer that three different chemotherapies did not stop? **(chemotherapy)** 9. To die in a nursing home? **(nursing)** 10. To die in a hospital? **(hospital)** 11. To die at home? **(home)**

**Appendix Table A2.** Right Answer Categories on the 11 End-of-life Medical Situations Questions

In your opinion, how likely is it in general in Switzerland…	Likelihood	References
To suffer from dementia at the age of 75 years **(dementia 75)**?	Very unlikely (0–25%)	*In Switzerland, the prevalence estimate of dementia is lower than 5% for individuals aged less than 75 years old and approximately 45% for adults of 95 years old.* ^23^
To suffer from dementia at the age of 95 **(dementia 95)**?	Rather unlikely (26%–50%)	
In the absence of advance directives, to be asked to make medical decisions concerning your spouse/partner should he/she become severely ill and unable to make decisions **(decisions)**?	Very likely (76%–100%)	*According to Swiss national law, the spouse/partner is the surrogate decision maker if the patient is unable to make decisions (Art 378 Swiss Civil Code).*
That pain (of any origin) can be successfully treated in a situation of imminent dying **(pain)**?	Very likely (76%–100%)	*In a recent population-based study on the last 30 days of life conducted in Canada, the authors found that although pain is a prevalent symptom at the end of life, less than 20% reported having severe daily pain.* ^10^
That artificial nutrition and hydration help in a situation of imminent dying **(artificial)**?	Very unlikely (0–25%)	*Artificial nutrition and hydration do not positively affect the dying phase.* ^27,28^
For a 70 years old to survive until hospital discharge from a cardiopulmonary resuscitation performed outside of a hospital after a cardiac arrest **(cardiopulmonary)**?	Very unlikely (0–25%)	*The survival rate for a 70 years old after a cardiopulmonary resuscitation is lower than 8%.* ^34^
That a hospital admission in the case of pneumonia helps for a patient in a situation of imminent dying due to advanced dementia living in a nursing home **(pneumonia)**?	Very unlikely (0–25%)	*A study showed that treating patients directly in their nursing homes with oral antibiotics was more beneficial for their quality of dying and considerably more cost-saving than hospitalization.* ^38^
That a fourth-line chemotherapy helps a patient with advanced cancer that three different chemotherapies did not stop **(chemotherapy)**?	Very unlikely (0–25%)	*Despite the increasing number of cancer survivors among the Swiss population, the likelihood that a fourth-line chemotherapy helps a patient is ∼13,6%.* ^39–41^
To die in a nursing home **(nursing)**?	Rather unlikely (26%–50%)	*According to a recent study, 44% of Swiss citizens aged 65 years and older die in a nursing home, 37% in a hospital, and 19% at home or elsewhere.* ^43^
To die in a hospital **(hospital)**?	Rather unlikely (26%–50%)	
To die at home **(home)**?	Very unlikely (0–25%)	
